# Rabbit as a Novel Animal Model for Hepatitis E Virus Infection and Vaccine Evaluation

**DOI:** 10.1371/journal.pone.0051616

**Published:** 2012-12-13

**Authors:** Xianfeng Cheng, Song Wang, Xing Dai, Chengbo Shi, Yufeng Wen, Ming Zhu, Shenwei Zhan, Jihong Meng

**Affiliations:** 1 Department of Microbiology and Immunology, School of Medicine, Southeast University, Nanjing, Jiangsu, China; 2 Department of Dermatology, Affiliated Zhongda Hospital, School of Medicine, Southeast University, Nanjing, jiangsu, China; 3 Changchun Institute of Biological Products Co. Ltd, Changchun, Jilin, China; 4 Department of Preventive Medicine, Wannan Medical College, Wuhu, Anhui, China; 5 Center for Disease Control and Prevention of Ma Anshan, Ma Anshan, Anhui, China; Kobe University, Japan

## Abstract

**Background:**

The identification of hepatitis E virus (HEV) from rabbits motivated us to assess the possibility of using rabbits as a non-human primate animal model for HEV infection and vaccine evaluation.

**Methodology/Principal Findings:**

First, 75 rabbits were inoculated with seven strains of genotypes 1, 3, 4, and rabbit HEV, to determine the appropriate strain, administrative route and viral dosage. Second, 15 rabbits were randomly divided into three groups and vaccinated with 0 µg (placebo), 10 µg and 20 µg of HEV candidate vaccine, HEV p179, respectively. After three doses of the vaccination, the rabbits were challenged with 3.3×10^5^ genome equivalents of genotype 4 HEV strain H4-NJ703. The strain of genotype 1 HEV was not found to be infectious for rabbits. However, approximately 80% of the animals were infected by two rabbit HEV strains. All rabbits inoculated with a genotype 3 strain were seroconverted but did not show viremia or fecal viral shedding. Although two genotype 4 strains, H4-NJ153 and H4-NJ112, only resulted in part of rabbits infected, another strain of genotype 4, H4-NJ703, had an infection rate of 100% (five out of five) when administrated intravenously. However, only two out of fifteen rabbits showed virus excretion and seroconversion when inoculated orally with H4-NJ703 of three different dosages. In the vaccine evaluation study, rabbits vaccinated with 20 µg of the HEV p179 produced anti-HEV with titers of 1∶10^4^–1∶10^5^ and were completely protected from infection. Rabbits vaccinated with 10 µg produced anti-HEV with titers of 1∶10^3^–1∶10^4^ and were protected from hepatitis, but two out of the five rabbits showed virus shedding.

**Conclusions/Significance:**

Rabbits may be served as an alternative to the non-human primate models for HEV infection and vaccine evaluation when certain virus strains, appropriate viral dosages, and the intravenous route of inoculation are selected.

## Introduction

Hepatitis E virus (HEV) is a major cause of enterically transmitted hepatitis worldwide [1], [2]. Epidemiology studies have showed that hepatitis E is widespread in developing countries [3]. In industrialized countries, sporadic hepatitis E has also been reported [4]–[6]. HEV is mainly transmitted by the fecal-oral route. Contaminated water can cause major epidemics in countries with poor sanitation conditions. In the past 10 years, increasing amounts of evidence suggested that zoonotic transmission may also be responsible for the spreading of HEV, especially in non-endemic areas [7], [8].

Previous studies have shown that rhesus monkeys and chimpanzees are susceptible to HEV of all known genotypes [9]–[11]. Studies also reported that swine can be used as an animal model for HEV genotypes 3 and 4 [12]–[14]. However, studies on the non-human primates and swine have been limited in the numbers of animals because of the high prices and difficulties in handling, manipulating, and housing them.

Recently, researchers have reported that HEV can be isolated from rabbits [15], [16]. The rabbit HEV strains shared about 73–77, 70–76, 75–82, 71–77, and 53–65% identity to the genotypes 1, 2, 3, 4 and avian HEV at the nucleotide level, respectively. These new discoveries broadened the known host ranges and diversity of the virus and suggest that rabbits could serve as an alternative animal model of HEV infection. The present report evaluated and analyzed the experimental infection of the rabbits with different genotypes of HEV strains by different administrative routes and different viral dosages. The protection provided by a HEV vaccine candidate (HEV p179) in the HEV-infected rabbit model was also evaluated herein.

## Materials and Methods

### Ethics Statement

All of the animals in this study were handled in a manner approved by the Committee of Laboratory Animal Welfare and Ethics, Southeast University (approval ID: 2009–036). The regulations issued by the review committee of laboratory animal welfare and ethics and the protocol for the review on laboratory animal welfare and ethics, Southeast University, were followed.

### Viruses

Seven HEV strains isolated from humans and rabbits were used in the present study. One strain of genotype 1 HEV was collected from the stools of patients with epidemic hepatitis E in Xinjiang, China (GenBank No: JX857689). A genotype 3 strain was collected from the stools of a US organ transplant recipient with chronic hepatitis E (GenBank No: JN837481). Three genotype 4 HEV strains, H4-NJ153 (GenBank No: JX847414), H4-NJ112 (GenBank No: JX847413), and H4-NJ703 (GenBank No: AY789228), were collected from the stools of patients with acute HEV infection in Nanjing, China. Two strains of rabbit HEV, R-JS174 (GenBank No: JQ065065) and R-JS204 (GenBank No: JQ065068), were recovered from stool samples representing secondary rabbit transmission (rabbit to rabbit).

The stool samples were diluted in phosphate-buffered saline (PBS, pH 7.4) containing 1% bovine serum albumin (BSA) to make 10% (wt/vol) suspensions. These suspensions were further clarified by centrifugation at 5,000 rpm at 4°C for 20 min and filtered through 0.22 µm filters. The quantification of HEV RNA in the samples was determined using a real-time RT-PCR kit (Liferiver, Shanghai, China).

### Animals

Ninety specific-pathogen-free (SPF) female New Zealand white rabbits, weighing 1.5–2.0 kg and aged 4–5 months, were purchased from the animal farm of Nanjing Agricultural University, Nanjing, China. The rabbits used in the present study were *Oryctolagus cuniculus* European rabbits. Prior to inoculation, all rabbits were tested for anti-HEV IgG antibodies with enzyme-linked immunosorbent assay (ELISA) and confirmed to be seronegative for HEV infection as described previously [17].

### Vaccine Candidate

The candidate hepatitis E vaccine, HEV p179, used in this study was expressed in *E.coli*-BL21(ED3)-*pLysS* strain (Novagen, Darmstadt, Germany) as described previously [18], [19]. It was produced by the Changchun Institute of Biological Products Co. Ltd, Chinese National Biotech Corporation (Changchun, China) in 10 µg/ml and 20 µg/ml formulated with alum adjuvant (1 mg/ml). The ability of HEV p179 to provide full protection from hepatitis was demonstrated in our previous study via a challenge test in non-human primates [data not shown].

### Experimental Design

As shown in Table 1, the present study included two experiments. In the first experiment, 40 rabbits were randomly divided into eight groups (groups H1, H3, R-JS204, R-JS174, H4-NJ703, H4-NJ153, H4-NJ112, and Control-1). The rabbits in all groups except for the control group were inoculated intravenously with different genotype HEV strains. Then, 35 rabbits were randomly divided into seven groups (groups iv-Low, iv-Med, iv-High, or-Low, or-Med, or-High, and Control-2) and the rabbits in all groups except for the control group were inoculated intravenously or orally with different doses of H4-NJ703 because this strain has shown itself capable of infecting rabbits efficiently in the first step. In order to infect rabbits with HEV via oral route, animals were orally administered with 1 ml of 10 mM sodium bicarbonate. One hour later, they were inoculated orally with different doses of the virus through rabbit stomach tubes.

**Table 1:** Design of the experimental infection and vaccine evaluation with rabbits inoculated with different HEV strains.

Experiment	Inoculum strain (Groups)	Virus Genotype	Infectious dose: Genome equivalents	Infection routes	Vaccine
1	H1	1	2.3×10^5^	iv	–
	H3	3	6.7×10^5^	iv	–
	R-JS204	Rabbit HEV	4.2×10^5^	iv	–
	R-JS174	Rabbit HEV	3.1×10^5^	iv	–
	H4-NJ703	4	3.3×10^5^	iv	–
	H4-NJ153	4	1.3×10^5^	iv	–
	H4-NJ112	4	2.2×10^5^	iv	–
	Control-1	–	0	–	–
2	H4-NJ703iv-Low	4	3.3×10^3^	iv	–
	H4-NJ703iv-Med	4	3.3×10^4^	iv	–
	H4-NJ703iv-High	4	3.3×10^5^	iv	–
	H4-NJ703or-Low	4	3.3×10^3^	or	–
	H4-NJ703or-Med	4	3.3×10^4^	or	–
	H4-NJ703or-High	4	3.3×10^5^	or	–
	Control-2	–	0	–	–
3	Vaccine-10	4	3.3×10^5^	iv	3×10 µg
	Vaccine-20	4	3.3×10^5^	iv	3×20 µg
	Placebo	4	3.3×10^5^	iv	3×0 µg

Five rabbits in each group. iv: intravenous inoculation, or: oral inoculation.

In the second experiment, 15 rabbits were randomly divided into three groups. Rabbits in group Vaccine-10 and group Vaccine-20 were vaccinated intramuscularly with 1 ml vaccine containing 10 µg and 20 µg HEV p179 vaccine, respectively, and five rabbits in another group received a placebo. Totally, three times of vaccination were administered by two weeks apart (0, 2, and 4 weeks). Two weeks after the third vaccination, all animals were challenged intravenously with 3.3×10^5^ genome equivalents of H4-NJ703.

### Sample Collection and Processing

Serum and stool samples were collected per week post inoculation (wpi) and stored at −80°C. Liver, bile, duodenum, jejunum, and ileum samples were collected at the end of the experiment (10 wpi). For the sample preparation, 0.2 g of different tissue sample was homogenized in 2 ml sterile PBS buffer (10% w/v) and clarified by centrifugation at 4°C at 3,000 rpm for 15 min. These samples were also stored at −80°C for the detection of HEV RNA. The liver tissue samples used for histopathologic examination were fixed in 10% neutral buffered formalin immediately upon sampling.

### Anti-HEV Antibody Detection

Anti-HEV IgG antibodies in rabbit sera were detected by an ELISA as described previously [17]. Briefly, microwell plates were coated with the purified protein p166mix overnight at 4°C. The samples were added to the wells with normal rabbit sera serving as negative controls. A colorimetric signal was developed with HRP conjugated goat anti-rabbit IgG and tetramethylbenzidine substrate. Reactions were terminated with 2 M sulfuric acid. The absorbance of each well was measured at 450 nm. The mean signal-to-cutoff (S/CO) values from each group at each wpi were calculated and values >1 were considered positive. The cut-off value was 0.152. It was determined using the mean optical density value of negative samples plus three standard deviations. The maximum dilution (starting at 1∶100) was defined as endpoint. Results were reported as geometric mean titers (GMTs). Reciprocal antibody levels <100 were assigned a value of 10.

### ALT Detection

All rabbits were monitored weekly for 10 weeks after inoculation. The sera were separated from clotted blood by low-speed centrifugation for 15 min at 4°C and the activities of alanine aminotransferase (ALT) were measured on the day of collection using an automated bio-chemistry analyzer (Beckman, CA, USA). ALT peak level exceeding two-fold of the base-line level was taken as biochemical evidence of hepatitis. This correlation was described in a previous study involving a non-human primate animal model [20].

### HEV RNA Detection

A universal RT-PCR assay was performed to detect HEV RNA in serum, stool, bile and liver tissue samples [21]. The universal RT-PCR assay is capable of detecting all 4 known genotypes of HEV and the rabbit HEV. Briefly, total RNAs were extracted using a RNeasy Mini Kit spin column (Qiagen, CA, USA) from 100 µl of the serum, fecal suspension, bile, or 10% tissue homogenate. The total RNAs were resuspended in 40 µl of DNase, RNase, and proteinase-free water. HEV was detected using reverse transcription and nested PCR with a set of universal HEV PCR primers. The external primers were JM-2 (forward, 5′-CCG ACA GAA TTG ATT TCG TCG GC) and JM-5 (reverse, 5′-CCG TAA GTG GAC TGG TCG TAC TC). The internal primers were JM-3 (forward, 5′-GTT GTC TCG GCC AAT GGC GAG CC) and JM-4 (reverse, 5′-TCG GCG GCG GTG AGA GAG AGC CA). Reverse transcription and first-round PCR were performed using a One-Step RT-PCR Kit (Qiagen, CA, USA). Reverse transcription was performed at 50°C for 45 min and terminated by heating at 95°C for 15 min, after which PCR continued for 35 cycles of denaturation at 94°C for 30 sec, annealing at 50°C for 30 sec, and extension at 72°C for 30 sec. This was followed by a final extension at 72°C for 10 min. Next, 3 µl of first-round PCR product was subjected to nested PCR using *Taq* DNA polymerase (Tiangen, Beijing, China) consisting of 35 cycles of denaturation at 94°C for 30 sec, annealing at 56°C for 30 sec, extension at 72°C for 30 sec. This was followed by a final extension at 72°C for 10 min. The amplicons were separated and visualized by electrophoresis on a 1% (wt/vol) agarose gel. The expected final product of the universal nested RT-PCR was 236 bp in length.

### Indirect Immunofluorescence Assay

Liver tissue sections, 7 µm in thickness, were derived from HEV infected rabbits and normal rabbits and then placed on slides and examined for HEV antigens using indirect immunofluorescence staining as reported previously [22]. Briefly, the monoclonal anti-HEV antibody 5G5, prepared in our laboratory, was added to sections and incubated at 37°C for 2 hours [23]. After washing with PBS, 1∶500 dilution of FITC-labeled goat anti-mouse secondary antibody (Santa Cruz, CA, USA) was added and incubated at 37°C for 30 min. After washing with PBS, slides were observed with a fluorescence microscope (Nikon, Tokyo, Japan).

### Histopathologic Examination

The liver tissues used for histologic examination were fixed in 10% neutral buffered formalin, routinely processed, sectioned at a thickness of 7 µm, and stained with hematoxylin and eosin. All sections were examined using an Olympus BH-2 microscope (Olympus, Beijing, China).

### Statistical Analyses

The mean and standard deviation, or frequency and percentage, were determined for the continuous variables and categorical variables, respectively. In the first experiment, the comparison of positive rates of the IgG anti-HEV antibody in different groups was conducted by Fisher’s exact test. In order to evaluate the infective ability of different HEV strains to rabbits, the trends of anti-HEV IgG antibodies and feces shedding at different wpi were also conducted by Fisher’s exact test in the present study. In the second experiment, the analysis of the variance was conducted to test the differences of the titers of anti-HEV IgG (GMTs) and the serum ALT levels in the different vaccine groups. Statistical analyses as above were performed with Statistical Product and Service Solutions 13.0 (SPSS; SPSS Inc, Chicago, USA).

## Results

### Infection of Rabbits Inoculated with HEV Strains of Different Geno- types

Rabbits from the control group did not show any clinical signs attributable to HEV infection and remained seronegative throughout the study (Table 2). Similar to the control group, no clinical symptoms of hepatitis were observed in group H1. Neither anti-HEV IgG nor viral shedding was detected in animals inoculated with the strain of genotype 1 HEV.

**Table 2:** Time course of seroconversion of rabbits inoculated with different genotype HEV strains.

Groups	Number of rabbits with seroconversion at indicated wpi										
	0	1	2	3	4	5	6	7	8	9	10
H1	0	0	0	0	0	0	0	0	0	0	0
H3	0	0	0	2	2	3	4	5	5	5	5
R-JS204	0	2	2	2	2	2	3	3	4	4	4
R-JS174	0	1	2	2	2	2	2	3	3	4	4
H4-NJ703	0	3	3	5	4	4	4	4	5	5	5
H4-NJ153	0	0	0	0	0	0	1	1	1	1	1
H4-NJ112	0	0	0	0	2	2	2	2	2	2	2
Control-1	0	0	0	0	0	0	0	0	0	0	0

Each group contained five rabbits.

In groups R-JS174 and R-JS204, 80% of the animals were seropositive and began to excrete virus one or two weeks after inoculation (Table 2 and 3). However, no clinical signs of HEV infection were observed in the animals from these two groups.

In group H3, all five rabbits were positive for anti-HEV IgG antibodies at the end of the experiment (Table 2). However, no clinical symptoms of hepatitis were observed and no viral shedding was detected in the rabbits from group H3.

One rabbit from group H4-NJ153 and two rabbits from group H4-NJ112 were antibody-positive but did not show any clinical signs of hepatitis. One rabbit from H4-NJ112 group was positive for anti-HEV-IgG and also positive for HEV RNA in a stool sample taken three weeks after inoculation. Viral shedding ceased one week later. The other animals remained negative for HEV RNA throughout the experiment.

In group H4-NJ703, all rabbits were anti-HEV-IgG-positive at the end of the experiment. Three of the five rabbits showed symptoms of hepatitis such as anorexia, lethargy, and diarrhea. All five animals in group H4-NJ703 excreted virus in their stool. The virus shedding began one or two weeks after intravenous inoculation, and persisted for three to ten weeks post inoculation (Table 3). Three rabbits in group H4-NJ703 showed mild, transient ALT elevations (2.4, 2.6 and 3.1-fold higher, respectively).

**Table 3:** HEV RNA detection in the stools of rabbits inoculated with different genotype HEV strains.

Groups	Number of rabbits with viral shedding in feces at indicated wpi										
	0	1	2	3	4	5	6	7	8	9	10
H1	0	0	0	0	0	0	0	0	0	0	0
H3	0	0	0	0	0	0	0	0	0	0	0
R-JS204	0	1	2	3	4	4	1	0	0	0	0
R-JS174	0	2	2	2	2	2	2	4	4	2	2
H4-NJ703	0	1	5	5	5	4	4	4	4	4	4
H4-NJ153	0	0	0	0	0	0	0	0	0	0	0
H4-NJ112	0	0	0	1	0	0	0	0	0	0	0
Control-1	0	0	0	0	0	0	0	0	0	0	0

Each group contained five rabbits.

**Figure 1:**
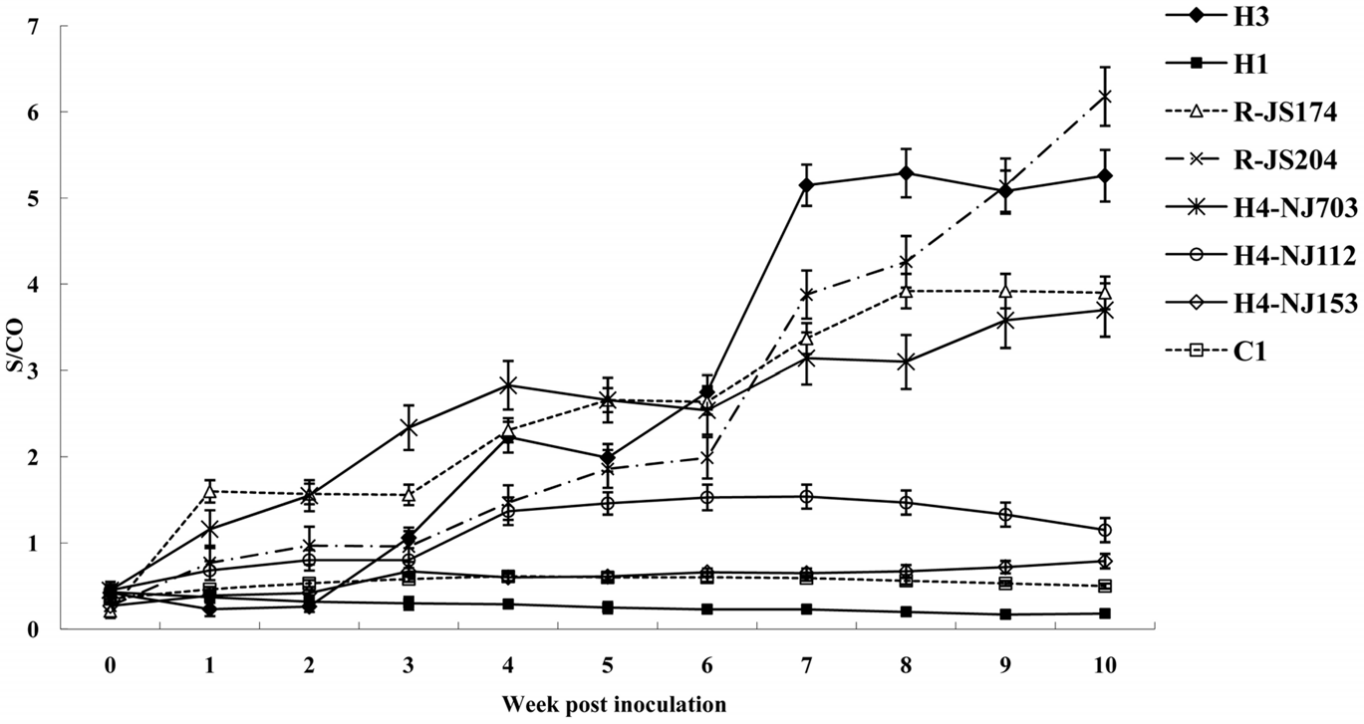
Time course of seroconversion of anti-HEV IgG in rabbits inoculated with HEV strains of different genotypes. The mean ELISA signal-to-cutoff (S/CO) values from each group at each wpi are plotted and used for the evaluating the trends of anti-HEV antibody developed by the animals inoculated with different genotype HEV strains. If the value is more than 1, the sample was considered as positive. The mean S/CO values in the groups H3, H4-NJ703, R-JS174, and R-JS204 increased gradually but were not statistically different from each other. However, the mean S/CO values in groups H1, H4-NJ153, H4-NJ112, and the control group did not increase.

The dynamic patterns of the mean S/CO values of anti-HEV antibodies of different groups were analyzed in Fig. 1. The changing trends of antibody levels based on the values in groups H3, H4-NJ703, R-JS174, and R-JS204 were similar and increased gradually by the end of the study, with the only discernable difference being the time to seroconversion (groups H3, H4-NJ703, R-JS174, and R-JS204 seroconverted at the 3^rd^, 1^st^, 1^st^ and 4^th^ week, respectively). However, the values in groups H1, H4-NJ153, H4-NJ112 and the control group did not increased.

### Infection of Rabbits Inoculated Intravenously or Orally with Different Doses of the H4-NJ703

**Figure 2:**
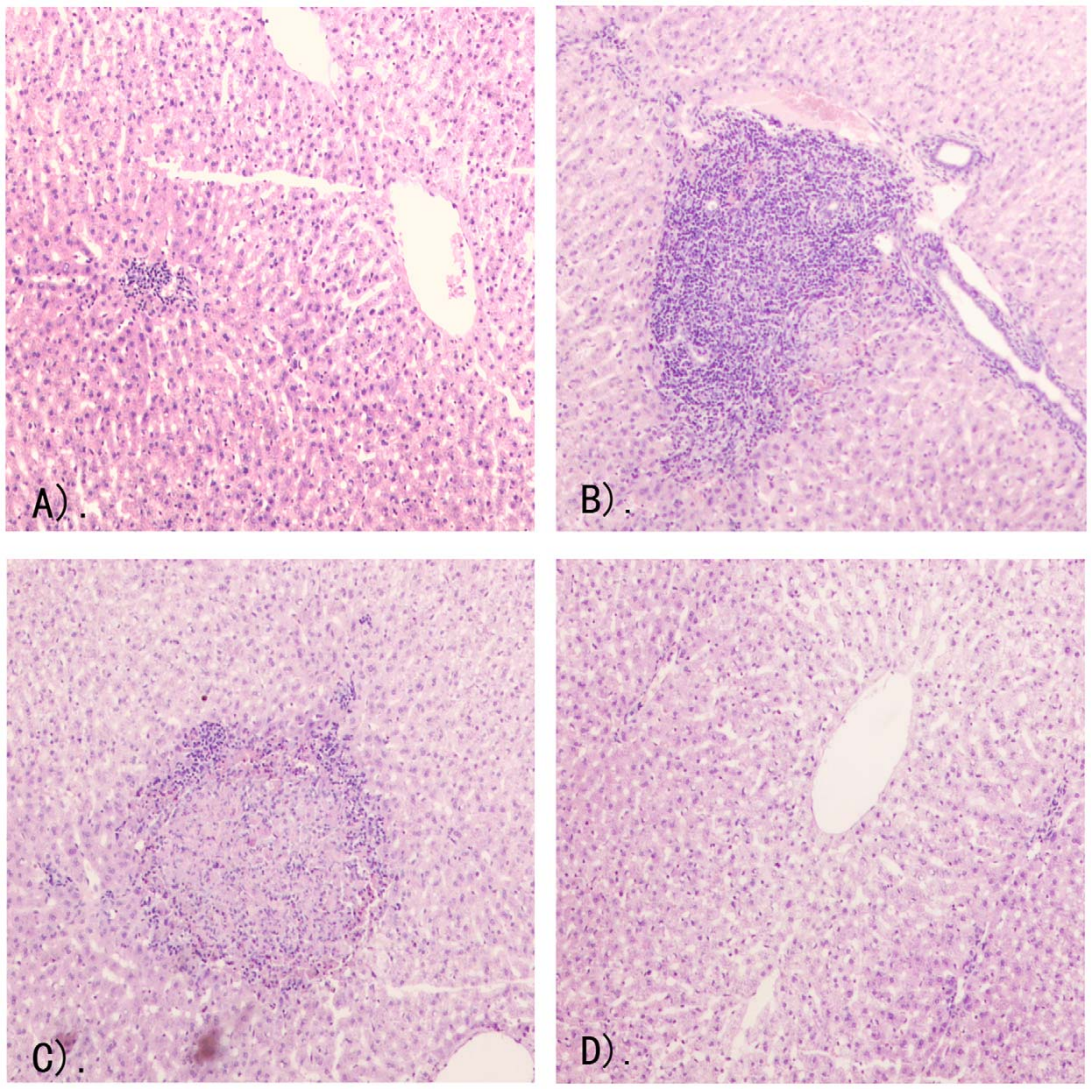
Pathological signs of HEV infection in hematoxylin and eosin stained liver sections. (A) Liver section from the rabbit in group iv-Low showing slightly distributed multifocal lymphohistiocytic infiltrates. (B) Liver section from the rabbit in group iv-Med showing accumulations of inflammatory cells. (C) Liver section from the rabbit in group iv-High showing localized hepatocellular necrosis. (D) Liver section from a control rabbit showing no visible pathological signs of HEV infection.

In the intravenous low dosage (iv-Low) group, in which the rabbits were inoculated with 3.3×10^3^ genome equivalents of H4-NJ703, no rabbits showed ALT elevation. Only two of five rabbits in this group showed fecal shedding and seroconversion. As shown in Fig. 2A, liver section from the rabbit in group iv-Low showed slightly distributed multifocal lymphohistiocytic infiltrates.

In group iv-Med, in which the rabbits were inoculated with 3.3×10^4^ genome equivalents of H4-NJ703, three animals shed virus in their feces and seroconverted to anti-HEV IgG antibodies. One of them also showed ALT elevation. Additionally, accumulations of inflammatory cells could be observed in liver section (Fig. 2B).

**Figure 3:**
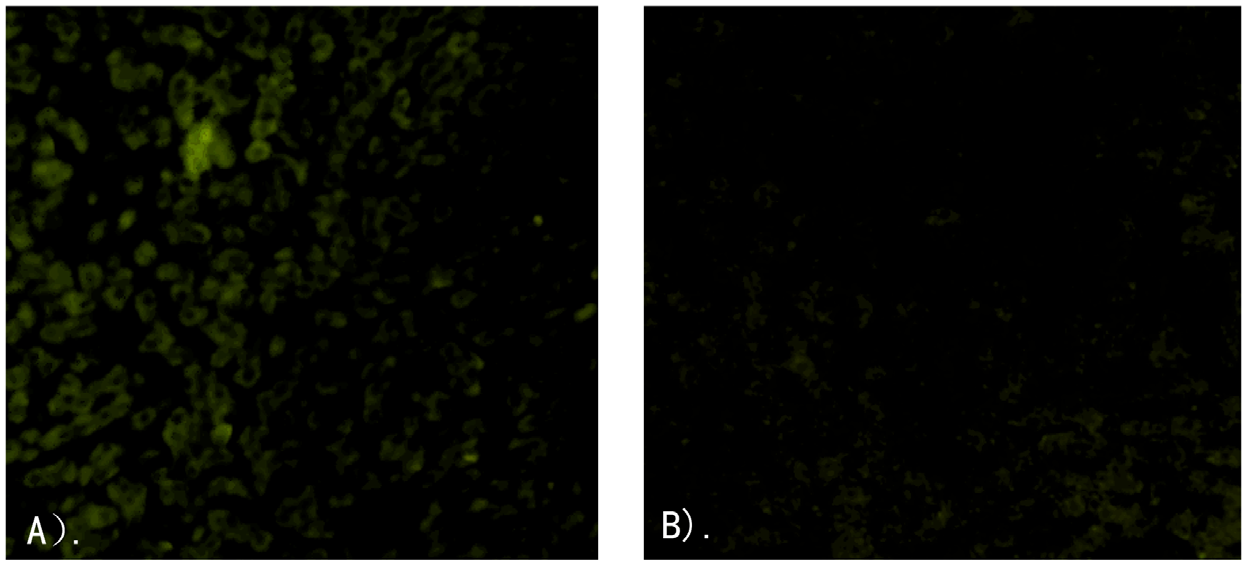
HEV antigen in liver cells of infected rabbits detected by indirect immunefluorescence. (A) Granular fluorescence of HEV antigens distributed in hepatocytes, ×100. (B) Liver of a rabbit from the negative control group, ×100.

In group iv-High, in which the rabbits were given the highest dosages of H4-NJ703 (3.3×10^5^ genome equivalents of virus), all rabbits presented one or more signs of HEV infection. One rabbit had detectable viremia, two had elevated ALT (2.1, 3.3-fold), and all had fecal shedding and seroconvertion (Table 4). Unlike the rabbits from group iv-Low and group iv-Med, the rabbits in iv-High showed multifocal lymphohistiocytic infiltrates distributed irregularly in the liver and local hepato- cellular necrosis in liver sections (Fig. 2C). Immunofluorescence staining indicated the presence of HEV antigens in the liver (Fig. 3A). Two rabbits in the iv-High group showed virus shedding two weeks after virus inoculation, and persisted for eight weeks. HEV RNA was detected in the bile, liver, and small intestines of these two rabbits.

**Table 4:** Experimental infection of rabbits with the H4-NJ703 strain by oral or intravenous route of inoculation.

Groups	Event	Number of positive rabbits tested at indicated wpi										
		0	1	2	3	4	5	6	7	8	9	10
Control-2												
	ALT elevation	0	0	0	0	0	0	0	0	0	0	0
	Fecal shedding	0	0	0	0	0	0	0	0	0	0	0
	Seroconversion	0	0	0	0	0	0	0	0	0	0	0
iv-Low												
	ALT elevation	0	0	0	0	0	0	0	0	0	0	0
	Fecal shedding	0	0	2	2	2	2	2	2	2	2	2
	Seroconversion	0	1	2	2	2	2	2	2	2	2	2
iv-Med												
	ALT elevation	0	0	1	1	1	1	1	0	0	0	0
	Fecal shedding	0	1	3	3	2	2	2	2	2	2	2
	Seroconversion	0	0	1	2	3	3	3	3	3	3	3
iv-High												
	ALT elevation	0	0	1	1	2	2	1	0	0	0	0
	Fecal shedding	0	1	4	5	5	3	3	2	2	2	2
	Seroconversion	0	3	4	5	5	4	4	5	5	5	5
or-Low												
	ALT elevation	0	0	0	0	0	0	0	0	0	0	0
	Fecal shedding	0	0	0	0	0	0	0	0	0	0	0
	Seroconversion	0	0	0	0	0	0	0	0	0	0	0
or-Med		0	0	0	0	0	0	0	0	0	0	0
	ALT elevation	0	0	0	0	0	0	0	0	0	0	0
	Fecal shedding	0	0	0	0	0	0	0	0	0	0	0
	Seroconversion	0	0	0	0	0	0	0	0	0	0	0
or-High		0	0	0	0	0	0	0	0	0	0	0
	ALT elevation	0	0	0	0	0	0	0	0	0	0	0
	Fecal shedding	0	1	2	2	0	0	0	0	0	0	0
	Seroconversion	0	2	2	1	1	2	2	2	2	2	2

Each group contained five rabbits.

The animals in oral groups showed a much lower rate of ALT elevation, fecal shedding, and seroconversion than those in the intravenous groups (p*<*0.05).

However, in the three oral groups, only two rabbits from group or-High showed virus excretion and seroconversion. The remaining rabbits showed no virologic or serologic evidence of HEV infection. The animals in these groups showed a much lower rate of ALT elevation, fecal shedding, and seroconversion than those in the intravenous groups (p*<*0.05).

According to the results, the dosage of 3.3×10^5^ genome equivalents of H4-NJ703 and the intravenous route of infection were selected for the HEV-infected-rabbit model development and used in the next experiment for the evaluation of the HEV vaccine candidate.

### Protection of the HEV p179 Vaccine in HEV-Infected Rabbit Model

Rabbits in groups Vaccine-10 and Vaccine-20 were immunized with 10 µg and 20 µg of the HEV p179 vaccine, respectively. In both groups, low-titer seroconversion occurred about two weeks after the first vaccination. However, the GMTs increased significantly after two booster immunizations at the 2^nd^ and 4^th^ week, reaching values of 10^3^–10^5^ after six weeks. The GMTs were significantly higher in the Vaccine-20 group than in the Vaccine-10 group (p<0.05) (Table 5).

**Table 5:** HEV p179 vaccine protects rabbits against challenge with the genotype 4 HEV.

[Group name] vaccine dosage	Rabbit code	Anti-HEV titer attime of challenge	ALT (peak/pre)	Virus shedding (weeks)	Viremia (weeks)
[Vaccine-10] 3×10 µg	1	1∶10000	1.8	0	0
	2	1∶10000	0.8	0	0
	3	1∶10000	1.1	0	0
	4	1∶1000	1.3	3	0
	5	1∶1000	1.5	3	0
[Vaccine-20] 3×20 µg	6	1∶10000	0.9	0	0
	7	1∶100000	1.2	0	0
	8	1∶100000	1.1	0	0
	9	1∶100000	0.8	0	0
	10	1∶100000	1.5	0	0
[Placebo] 3×0 µg	11	<1∶100	1.67	3	0
	12	<1∶100	2.55	8	0
	13	<1∶100	4.42	5	0
	14	<1∶100	3.32	10	1
	15	<1∶100	1.19	8	0

Pre-infection HEV antibody levels, expressed in titers, were significantly higher in the Vaccine-20 group than in the Vaccine-10 group (p<0.05). The frequency of animals having peak/pre-infection ALT >2.0 in the Placebo group was significantly higher (p<0.05) than the two vaccinated groups. The frequency of animals excreting the virus in the stools was significantly higher (p<0.05) in the control animals than the vaccinated animals, but was similar between the two groups of vaccinated animals.

After viral challenge, three of the five rabbits in the unvaccinated control group showed a significant elevation in serum ALT, as indicated by a peak ALT level exceeding two-fold of the corresponding base-line level (2.55, 4.42 and 3.32-fold, respectively). All of the control animals excreted virus in their stools. This was first detectable one week after infection and continued for three to ten weeks. The vaccine inhibited ALT elevation when the animal in groups Vaccine-10 and Vaccine-20 were challenged by H4-NJ703. No animal in group Vaccine-20 showed HEV excretion in their feces but two of five rabbits in group Vaccine-10 did (Table 5).

## Discussion

Several types of animal models for HEV infection have been identified, characterized, and refined in the previous studies [9], [12], [24]–[28]. Generally, non-human primates such as cynomolgus and rhesus monkeys are the best known models because they can be infected with HEVs of different genotypes and the course of HEV infection is similar to that in humans. SPF swine have also been successfully infected with genotypes 3 and 4 HEV from humans. However, the limited availability and high cost of primates and swine, as well as the difficulties in handling them, has severely restricted their use in large numbers. Recently, HEV RNA and anti-HEV antibodies have been detected in rabbits from two farms in China’s Gansu Province [15]. One study showed that rabbits could be experimentally infected with both rabbit HEV and genotype 4 of human HEV [27]. These results suggest that rabbits might be a suitable model for HEV infection and vaccine evaluation.

In the present study, rabbits were successfully infected with H4-NJ703 (human HEV genotype 4), as indicated by viral shedding in the stools, characteristic histopathological changes, and elevation in the level of liver enzyme. However, the infectivity of H4-NJ153 and H4-NJ112 to rabbits was much lower than that of H4-NJ703 although all these three strains are in the same genotype. In this way, these results indicated that although different HEV strains had very similar genomic organization, they could vary significantly in their ability to infect rabbits. Such results were also found in previous studies [9], [10], [28]–[31]. Rhesus and cynomolgus monkeys, although widely used as animal models of HEV infection, showed variations in levels of virus excretion, liver enzyme elevation, and histopathologic changes in liver when inoculated with different HEV strains [9], [10], [28]. In addition, Meng, *et al* and Krawczynski, *et al* found that swine could be used as an animal model for HEV genotypes 3 and 4 but not for genotypes 1 or 2 [29], [30]. Swine infected with genotype 3 human HEV developed more severe and persistent hepatic lesions than those infected with swine HEV [31]. Previous results from rabbits showed genotype 1 and 4 HEV to be ineffective in infecting rabbits [27]. However, our results showed the strain H4-NJ703 of genotype 4 to be a strong infectious agent in rabbits. Therefore, in order to develop a satisfactory HEV-infected rabbit model, further studies should be carried out to compare the infective ability of different HEV strains.

Although the fecal-oral route is the most common route of HEV transmission, almost all experimental studies on HEV have used intravenous inoculation as the route of infection because the former is inefficient [32]. In this case, even high doses of 10^5^ genome equivalents was insufficient to induce a high infection rate in three oral groups because only two out of the five inoculated rabbits showed some measurable infection (Table 4).

In this study, nearly all of the placebo-treated rabbits intravenously challenged with 3.3×10^5^ genome equivalents of H4-NJ703 showed fecal virus shedding and biochemical evidence of hepatitis. Two doses of candidate HEV p179 vaccine, at either 10 µg or 20 µg per dose, were highly effective in preventing ALT elevation following the challenge with HEV. However, although no animal in group Vaccine-20 showed HEV RNA excretion in the feces, two of five rabbits in group Vaccine-10 did. This was consistent with previous reports [33], [34]. In one study, although all monkeys in the vaccinated groups were protected against hepatitis E disease (as indicated by hepatic biochemistry), they were not well-protected against HEV infection (as indicated by viral excretion) [33]. In another study, three of nine rhesus monkeys vaccinated with a 5 µg and 10 µg dosages of HEV 239 vaccine showed the HEV excretion in their stool. However, the duration of viral excretion was significantly shorter in vaccinated animals than in placebo animals [34].

The major implication of this work is that rabbits can serve as an alternative animal model for the study of HEV. The present study also has several limitations. First, although the rabbits in group H3 became positive to anti-HEV antibodies, no fecal shedding of HEV RNA and no elevations of serum ALT were found in these animals. In order to assess the infectivity of genotype 3 HEV in rabbit, more different genotype 3 HEV strains should be studied. Second, in order to compare the ability of the infectivity of different HEV strains in rabbits, methods to titrate the active viral particles in the inoculum must be developed. Third, the present data indicated significant differences in the duration of viral excretion among different animals in the same group. In this way, the interactions between the virus and rabbits, including the mechanisms underlying HEV infection in rabbits should be further studied.

In summary, the present study demonstrates that rabbits may serve as a non-primate small animal model for HEV infection and vaccine evaluation when certain virus strains, appropriate viral dosages, and the intravenous route of inoculation are selected.
